# Resources and tools for rare disease variant interpretation

**DOI:** 10.3389/fmolb.2023.1169109

**Published:** 2023-05-10

**Authors:** Luana Licata, Allegra Via, Paola Turina, Giulia Babbi, Silvia Benevenuta, Claudio Carta, Rita Casadio, Andrea Cicconardi, Angelo Facchiano, Piero Fariselli, Deborah Giordano, Federica Isidori, Anna Marabotti, Pier Luigi Martelli, Stefano Pascarella, Michele Pinelli, Tommaso Pippucci, Roberta Russo, Castrense Savojardo, Bernardina Scafuri, Lucrezia Valeriani, Emidio Capriotti

**Affiliations:** ^1^ Department of Biology, University of Rome Tor Vergata, Roma, Italy; ^2^ Department of Biochemical Sciences “A. Rossi Fanelli”, University of Rome “La Sapienza”, Roma, Italy; ^3^ Department of Pharmacy and Biotechnology, University of Bologna, Bologna, Italy; ^4^ Department of Medical Sciences, University of Torino, Torino, Italy; ^5^ National Centre for Rare Diseases, Istituto Superiore di Sanità, Roma, Italy; ^6^ Department of Physics, University of Genova, Genova, Italy; ^7^ Italiano di Tecnologia—IIT, Genova, Italy; ^8^ National Research Council, Institute of Food Science, Avellino, Italy; ^9^ Medical Genetics Unit, IRCCS Azienda Ospedaliero-Universitaria di Bologna, Bologna, Italy; ^10^ Department of Chemistry and Biology “A. Zambelli”, University of Salerno, Fisciano, SA, Italy; ^11^ Department of Molecular Medicine and Medical Biotechnology, University of Naples Federico II, Napoli, Italy; ^12^ CEINGE Biotecnologie Avanzate Franco Salvatore, Napoli, Italy; ^13^ Center for Technology and Innovation, Trieste, Italy

**Keywords:** rare disease, genetic disorder, single nucleotide variant (SNV), genome interpretation, precision medicine, genotype-phenotype association, machine learning

## Abstract

Collectively, rare genetic disorders affect a substantial portion of the world’s population. In most cases, those affected face difficulties in receiving a clinical diagnosis and genetic characterization. The understanding of the molecular mechanisms of these diseases and the development of therapeutic treatments for patients are also challenging. However, the application of recent advancements in genome sequencing/analysis technologies and computer-aided tools for predicting phenotype-genotype associations can bring significant benefits to this field. In this review, we highlight the most relevant online resources and computational tools for genome interpretation that can enhance the diagnosis, clinical management, and development of treatments for rare disorders. Our focus is on resources for interpreting single nucleotide variants. Additionally, we present use cases for interpreting genetic variants in clinical settings and review the limitations of these results and prediction tools. Finally, we have compiled a curated set of core resources and tools for analyzing rare disease genomes. Such resources and tools can be utilized to develop standardized protocols that will enhance the accuracy and effectiveness of rare disease diagnosis.

## 1 Introduction

The recent major advances in genome sequencing and analysis technology have opened the road to exome and genome sequencing (ES/GS) as a common diagnostic tool for individual patients (Turro et al., 2020; 100,000 Genomes Project Pilot Investigators et al., 2021). Especially in the field of rare genetic diseases, the use of ES/GS has brought unprecedented progress, and holds the potential for further large-scale impact in the clinical setting, allowing early diagnosis and early, precisely tuned, treatment (Pogue et al., 2018; Liu et al., 2019; Claussnitzer et al., 2020; Bonne, 2021). Presently, the definition of a rare disease (RD) varies among different regions. In Europe, it is defined as a condition affecting not more than 1 person per 2,000 in the European population (Regulation Orphan Medicinal Product, 2000). In the United States, it is defined as a condition that affects less than 200,000 people in the country (U.S. Food and Drug Administration, 2022), while in Japan it is defined as affecting fewer than 50,000 people, or one in 2,500 (Hayashi and Umeda, 2008). Collectively, RDs represent a significant burden to health and society, as their estimated prevalence is approximately 3.5%–5.9% of the worldwide population, resulting in about 30 million people affected in Europe and 300 million worldwide (Nguengang Wakap et al., 2020). Approximately 7,000 different RDs have been identified to date, even though the exact number is debated (Hartley et al., 2018; Ferreira, 2019; Haendel et al., 2020), of which an estimated 70% are genetic (with 4,418 involved genes identified so far, November 2022), whilst the remaining are the results of infections, allergies and environmental causes. Most likely, the number of involved genes is bound to increase, as rapidly increasing quantities of exomic data are analyzed in the clinic (Boycott et al., 2018; 2019). From 2010 to 2020, the diagnosis of RDs saw a remarkable increase, with 886 new RDs being identified. During this period, the total number of genes associated with RDs grew from approximately 2,400 to over 4,000, and the number of new orphan drugs approved by the US and/or the European Union rose to 438 (Monaco et al., 2022).

Due to the very status of being rare, knowledge, research, medical expertise, and therapeutic opportunities for each particular RD are often extremely limited, and geographically sparse. Along with technological advances, the public and scientific awareness has been growing, and the knowledge on RDs is going to massively benefit from large scale data collection, integration, and sharing (Hartley et al., 2020). Many international initiatives and consortia (Gainotti et al., 2018; Azzariti and Hamosh, 2020; Bonne, 2021; Baxter et al., 2022; Laurie et al., 2022; Monaco et al., 2022) aim to significantly increase the overall percentage of RD patients with a confirmed (molecular) diagnosis, estimating that thousands of RD genes and disease mechanisms still remain undiscovered (Frésard and Montgomery, 2018; Boycott et al., 2019; Hartin et al., 2020). Exome Sequencing (ES) has been the most significant technology driving progress in the discovery and diagnosis of RDs over the past decade. While some RD diagnoses may require the integration of multiple omics data (Frésard and Montgomery, 2018; Marwaha et al., 2022), it is expected that ES will continue to play a crucial role in future efforts (Boycott et al., 2019).

The sheer re-analysis of exomic data after 1–3 years updating of the major disease variants and disease-gene association databases is reported to have increased the diagnosed cases by over 10% (Wenger et al., 2017; Setty et al., 2022). Remarkably, a further improvement in the yields could be obtained by reanalysing the data in collaboration with the clinician who made the diagnosis (Basel-Salmon et al., 2019). The contribution of research laboratories has provided an additional increase, aided by the application of novel computational and analysis tools (Eldomery et al., 2017). Thus, the fundamental step in ES data processing is the interpretation of the identified variations, i.e., the estimate of their likelihood of having a causative role in contributing to the disease. Indeed, RD-affected individuals often carry multiple variations in the gene(s) associated with the disease, with only a fraction of them being actually pathogenic (Summers, 1996). Criteria for the objective classification of variants into a five-tier system (pathogenic/likely pathogenic/uncertain significance/likely benign/benign) have been provided to the biomedical community, together with scoring rules that weight each criterion used to classify the variants. In this context, computational tools have a role in supporting the evidence framework for a benign or a pathogenic assertion (Richards et al., 2015).

This paper aims to provide an updated overview of the most frequently adopted and publicly accessible online resources and computational tools for predicting genotype-phenotype associations in RDs. In the first part of this review, we focus on the main databases collecting genes and variants associated with RDs. In addition, we describe the most popular computational methods for gene and variant prioritization, showing how information derived from molecular databases and tools can improve the diagnosis of RDs in clinical settings. Finally, we discuss the central role of FAIR data sharing in boosting research and diagnosis in the field and provide future perspectives.

## 2 Online resources and databases for rare diseases

Large-scale sequencing efforts on healthy individuals and patients allowed the collection of large databases of genetic variants and their association with human phenotypes. Based on their content and purposes, two groups of online resources for RDs can be identified: one group includes databases that define phenotype ontologies and controlled vocabularies for the description and classification of human diseases and phenotypes; the second group includes databases collecting the frequency of variants in the human population and their relationship with genetic disorders. Here, we summarize the most popular resources for medical diagnosis, focusing specifically on those related to RDs.

### 2.1 Disease and phenotype classification databases and ontologies

Nowadays, different resources for the classification of RDs are available. In particular, specific ontologies based on controlled vocabularies are defined for the description of human disorders. This enables a standardized description and classification of RDs, thereby enhancing and supporting data sharing. A standardized medical terminology was defined for developing the Medical Subject Headings (MeSH), an organized collection of hierarchical trees with increasing specificity of the downstream terms (Rogers, 1963). Later, ontologies based on diagnostic terms were created. Among them, the International Classification of Diseases (ICD), which represents the healthcare classification system maintained by the World Health Organization (World Health Organization, 2019), and the Systematized Nomenclature of Medicine (SNOMED), which implements a directed acyclic graph architecture for the automatic exploration of relationships among terms. In the 80s, the US National Library of Medicine created the Unified Medical Language System (UMLS) to harmonize the various classification systems (Bodenreider, 2004). ULMS, with its well-defined semantic relationships, is widely recognized as one of the most comprehensive resources for determining disease similarity and for the harmonization of RD data (Zhu et al., 2020). The increasing popularity of controlled vocabularies for the classification of human disorders further stimulated the creation of disease- and phenotype-oriented ontologies. The Human Phenotype Ontology (HPO) is a standardized vocabulary describing phenotypic abnormalities (Robinson et al., 2008). It is structured as a directed acyclic graph, in which a child node corresponds to a more specialized term with respect to its parent. Currently, HPO contains over 13,000 terms and over 156,000 annotations to hereditary diseases. The Disease Ontology (DO) is an open source ontology for the integration of biomedical data associated with human disease. The DO integrates concept terms from SNOMED, ICD, MeSH, and UMLS, using various semantic similarity measures (Schriml et al., 2012). The current version of DO (August 2022) collects more than 11,000 disease terms divided in 6 major classes. Mondo is the disease ontology of the Monarch Initiative (Shefchek et al., 2020) which integrates genotype-phenotype data across different species. The Mondo ontology is an open, community-driven resource which currently collects ∼44,650 terms divided in three main categories (disease characteristic, disease or disorder, disease susceptibility). In Mondo, human diseases are grouped in 36 classes. Biomedical ontologies serve various purposes, such as: 1) systematizing the description of biomedical concepts for literature and clinical data recording (e.g., MeSH), 2) capturing individual clinical phenotypes, even in the absence of a recognized disease, and providing a corresponding classification in animal models (e.g., HPO), and 3) categorizing nosological entities for epidemiological and clinical management purposes (e.g., ICD). There is often overlap between these classifications, with some incorporating features from others. These ontologies of concepts are also utilized to annotate molecular data databases for the purpose of storing, analyzing, and exploring genotype-phenotype relationships.

**FIGURE 1:**
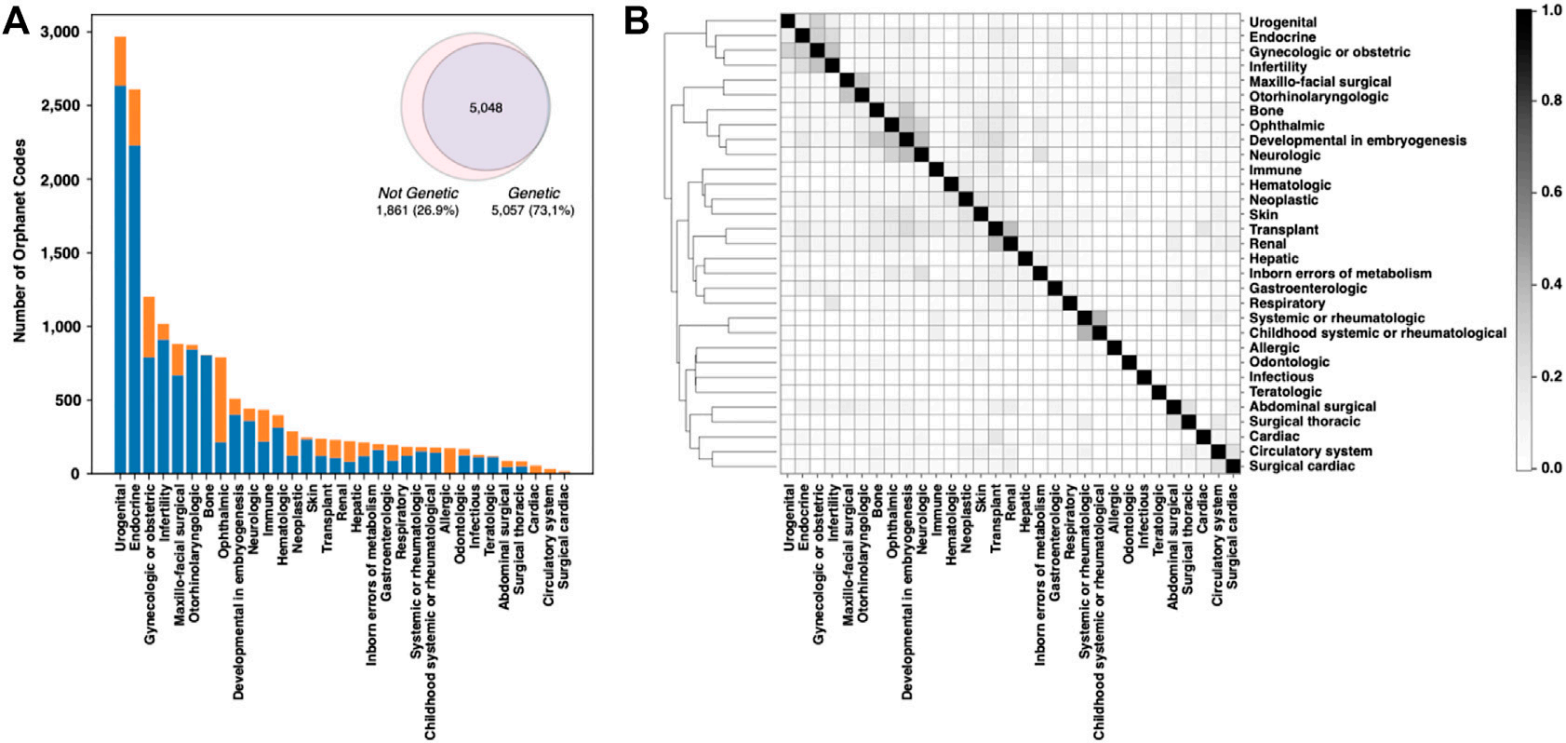
Analysis of the Orphanet database composition. **(A)** Fraction of genetic and nongenetic RDs in the different classes. **(B)** Plot showing the fraction of genes shared by each RD-class pair. Genes and Orphanet codes can be found to be associated with multiple RD classes.

The Online Mendelian Inheritance in Man (OMIM) database was created in the 60s by McKusick to systematically identify the relationship between disease and genetic components (Amberger et al., 2009). In November 2022, OMIM collected 7,301 phenotypes, ∼85% of which were associated with at least one of the 4,674 listed genes. Focusing on the classification of rare human disorders, Orphanet is a unique resource that provides high-quality information for defining a specific nomenclature for RD (Rath et al., 2012). The description of RDs in Orphanet is based on the Orphanet Rare Disease Ontology (ORDO), a structured vocabulary capturing relationships between diseases, genes, and other relevant features. The November 2022 version of the Orphanet database collects 6,918 RDs classified in 33 major groups. All groups include genetic-caused RD, except for the “toxic effects” group. The fraction of rare genetic diseases across the remaining 32 major classes ranges from 99.6% of the *“rare inborn errors of metabolism”* to the lowest percentage of *“infectious”* diseases. The most abundant types of rare genetic disorders are those having *“developmental”* and *“neurologic”* effects (Figure 1A). Overall, RDs with genetic origin represent ∼73% of the total (Figure 1A, inset). In terms of RD-associated genes, Orphanet collects more than 4,400 genes. Several of those genes are found to be associated with more than one RD class. An index of similarity (Jaccard index), based on the fraction of shared genes, has been calculated between each pair of RD groups, and is plotted in Figure 1B. The groups of “neurological” and “developmental” RD are sharing the highest number of disease-associated genes, with a Jaccard index ∼0.37. The full list of the fraction of genetic RD and associated genes is reported in Supplementary Table S1.

In terms of enzymatic function, out of 5,057 genetic RDs reported in Orphanet (Supplementary Table S1), 1,596 (31.6%) are associated with enzymes, distributed among all the seven major enzyme classes (Table 1). The most represented enzyme classes are Transferases, Hydrolases, and Oxidoreductases. Orphanet RDs can be linked to their corresponding enzyme metabolic pathways through the Reactome database, a comprehensive resource that catalogs all human metabolic reactions in 2,580 hierarchically organized pathways, with 27 main roots. Table 1 shows, for each enzyme class, the number of enzymes involved in Orphanet RDs, the number of Orphanet diseases that involve those enzymes, their Reactome roots and pathways.

**TABLE 1:** For each enzyme class, the table lists the number of enzymes associated with Orphanet RDs, the number of the corresponding Orphanet diseases, and the number of the corresponding Reactome roots and pathways. The data were derived from DAR database (Savojardo et al., 2022) that integrates gene-disease associations reported in UniProt, Monarch, and ClinVar.

Enzyme class	Enzymes^a^	Orphanet diseases^b^	Reactome roots	Reactome pathways
All classes	1,218	1,596	27	1,098
EC 1: Oxidoreductases	186	259	20	209
EC 2: Transferases	474	738	26	799
EC 3: Hydrolases	401	611	27	592
EC 4: Lyases	63	81	15	93
EC 5: Isomerases	40	62	17	78
EC 6: Ligases	58	76	7	28
EC 7: Translocases	44	77	11	36

^a^ In the distribution among classes, multiclass enzymes are counted multiple times.

^b^ In the distribution among classes, diseases associated with enzymes from different classes are counted multiple times.

The Disease And Reactome (DAR) database (Savojardo et al., 2022) provides a wealth of information on enzymes, including their relationships with Reactome pathways, molecular interactions within the pathways, and tissue expression levels as recorded in the Human Protein Atlas (Uhlén et al., 2015).

In general, the evaluation of the evidence supporting gene-disease relationships is a critical factor for an accurate diagnosis (Strande et al., 2017). To prevent mistakes in the diagnostic process, the curators of the Clinical Genome Resource (ClinGen) (Rehm et al., 2015) defined evidence-based Standard Operating Procedures for the classification of clinically relevant genes based on the presence of pathogenic variants (Section 1.3). Gene-disease relationships are classified in six groups that qualitatively describe the strength of the supporting evidence. The default class assigned to genes without any detected disease-causing variants is *“No Reported Evidence”*. Supporting evidence for gene-disease relationships is classified into four categories: *“Limited”*, *“Moderate”*, *“Strong”* and *“Definitive”*. When both supporting and conflicting evidence are present, the gene-disease relationship is classified as *“Contradictory”*. Within the ClinGen framework, the systematic review of genetic, clinical and experimental evidence, reported in databases such as OMIM and Orphanet, is used to assign one of the categories mentioned above to the reported gene-disease relationship.

### 2.2 Gene and protein network databases

A single gene defect is the most common origin of rare genetic diseases collected in the databases mentioned above. However, to investigate the molecular mechanisms underlying a RD, it is fundamental to understand and contextualize the resulting phenotype. At the protein level, defining the macromolecular complexes and pathways perturbed by the defective gene can be a useful strategy to understand the pathology itself and to intervene to restore the healthy phenotype.

A genetic variant can impact protein function and, depending on the central or marginal role of the mutated node inside a protein-protein interactions network, also the capability of the network to find alternative paths in the edges map. Changes in specific interactions can drastically perturb cellular networks and generate disease phenotypes (Barabasi et al., 2011; Menche et al., 2015).

Molecular interactions, mostly protein-protein interactions (PPIs), are annotated and archived, in structured formats, into several public resources. The major public databases collecting molecular interaction data can be divided into primary, predictive and meta-databases. Primary databases collect only manually curated molecular interactions, extracted from peer-reviewed journals, such as the IMEx Consortium resources (MINT (Calderone et al., 2020), IntAct (Del Toro et al., 2022), DIP (Salwinski et al., 2004), MatrixDB (Clerc et al., 2019)), and BioGRID (Oughtred et al., 2021). Meta-databases integrate data coming from primary databases, such as HiPPIE (Alanis-Lobato et al., 2017) and mentha (Calderone et al., 2013). Predictive databases use computational methods to predict PPIs (De Las Rivas and Fontanillo, 2012), such as STRING (Szklarczyk et al., 2021), IID (Pastrello et al., 2020) or ProfPPIdb (Tran et al., 2018).

In the panorama of molecular interaction resources, only the IMEx Consortium databases annotate interaction associated features, such as binding sites involved in the interaction or mutation effects (Porras et al., 2020). In particular, the IMEx mutation dataset contains annotations of experimental evidence where mutations have been shown to affect a protein interaction (∼75,000 records) (IMEx Consortium Curators et al., 2019). The dataset can be used to map selected pathogenic variants to manually curated PPIs and to understand the effect of a specific variant on the interactions at protein-protein interface. Moreover, from the IntAct datasets, it is possible to download a RD specific dataset of molecular interactions extracted from literature. The dataset is enriched with experimentally proven impact of clinical mutations on interactions, and also with the non-clinical mutations which are found to impact protein functionality. So far, the dataset contains over 7,900 interactions involving about 2,500 interactors. The dataset can be visualized and filtered in the IntAct result page, or in Cytoscape (Shannon et al., 2003), using the IntAct App (Ragueneau et al., 2021).

Disease specific biological networks can also be constructed or integrated with data coming from signaling pathways databases such as Signor (Lo Surdo et al., 2023), WIKIPathway (Martens et al., 2021) or OmniPath (Türei et al., 2016). They can then be imported into Cytoscape by using resource specific CytoscapeApps (Kutmon et al., 2014; Ceccarelli et al., 2020; De Marinis et al., 2021), to gain more insight into the molecular mechanisms involved in the disease. Moreover, pathway resources such as KEGG (Kanehisa et al., 2017) and Reactome (Jassal et al., 2020) databases are very important to discover whether some disease-associated subnetworks are enriched for a particular functional pathway.

By the combination of PPI with genotype-phenotype relationships, functional similarities have been used to generate specific disease networks defining similarity across different human disorders (Goh et al., 2007; Menche et al., 2015; Buphamalai et al., 2021). Such networks have been shown to be useful for studying the biological mechanisms of diseases and for the development of gene prioritization tools (Zhang and Itan, 2019). Some examples of gene prioritization tools, specific for RDs, will be discussed in Section 2.2.

### 2.3 Databases of variants

The Human Genome Variation Society (HGVS) maintains comprehensive lists of databases focused on variations, from locus-specific mutation databases to SNP databases, to chromosomal variations, to other mutation databases, including nonhuman and artificial mutations. However, given the high number of resources, it is nearly impossible to perform an exhaustive description of all those that are available. We will therefore focus on selected, curated and widely used resources. None of them is specifically dedicated to RDs; however, it is possible to collect data and information on RD-associated variations.

In general, variant databases can be divided into two groups, according to whether they focus on the variant’s frequency across the human populations or on their pathogenic effect.

The variant’s frequency can be derived from sequencing experiments on a large set of individuals. For example, the 1,000 Genome project, started in 2008, collected and sequenced the genomes of 2,504 individuals from 26 populations worldwide, characterizing more than 88 million variants, including >99% of SNP variants with a frequency higher than 1% (1000 Genomes Project Consortium et al., 2015). The datasets and the related analyses have been freely shared with the scientific community by setting up the International Genome Sample Resource (IGSR) (Fairley et al., 2020) to ensure their future usability and accessibility. Data about these variants can be explored through the Ensembl Variation database (Hunt et al., 2018), a project aimed at automatically annotating the genomes, integrating biological data and making all information accessible via a website. Those variants were grouped into subsets, based on the origin of the individual and on the frequency of occurrence. In the same period, the UK10K project (UK10K Consortium et al., 2015) sequenced the whole genomes of about 10,000 individuals, characterizing over 24 million novel sequence variants. That information was made available via a dedicated website and via the European Genome-phenome Archive (EGA) (Freeberg et al., 2022), a resource for permanently archiving and sharing personally identified genetic, phenotypic and clinical data, obtained by biomedical research projects. Another analogous study is the “All of Us'' research program, funded by NIH, sequencing 100,000 genomes from ethnic groups underrepresented in previous projects (All of Us Research Program Investigators et al., 2019). While the “All of Us” project was of broader scope, the 100,000 Genomes Project, focused on patients with an RD (161 disorders covering a broad spectrum of RDs were present) or with one among 20 different common cancer types (Turnbull et al., 2018). A pilot study, conducted on the genomes of 4,660 people, increased the diagnosis number for 25% of participants. Among them, 14% of the cases were new diagnoses based on variants found in regions usually missed in conventional, non-whole genomic tests (100,000 Genomes Project Pilot Investigators et al., 2021).

A widely used database collecting variant frequency data is the Genome Aggregation Database (gnomAD). The gnomeAD is the successor to the Exome Aggregation Consortium (ExAC), a project that was launched to aggregate and harmonize exome and genome sequencing data from a variety of large-scale sequencing projects (Karczewski et al., 2020). The National Center for Biotechnology Information (NCBI) at the NIH hosts several resources for investigating and understanding human variations. dbSNP and dbVar are two freely available databases, the former hosting a broad collection of small genetic polymorphisms (SNP, deletion/insertion polymorphisms, etc.), and the latter hosting a broad collection of large variants (>50 bp) (Lappalainen et al., 2013).

The second class of variant databases collect information about their clinical significance and their association with human disorders. To this purpose, the American College of Medical Genetics and Genomics and the Association for Molecular Pathology (ACMG/AMP), developed specific guidelines, where variants are classified into five types: *“pathogenic”*, *“likely pathogenic”*, “uncertain significance”, *“likely benign”*, and *“benign”* (Richards et al., 2015). On the one hand, *“pathogenic”* and *“likely pathogenic”*, variants are classified by using multiple criteria grouped in four weighted categories: *“very strong”*, *“strong”*, *“moderate”*, and *“supporting”*. On the other hand, *“likely benign”*, and *“benign”* variants are classified using a combination of rules grouped in three weighted categories: *“stand-alone”*, *“strong”* and *“supporting”*. All previous criteria are based on eight categories of information, including among them data from population studies and computational predictions. If a variant does not meet any criteria or the evidence for benign and pathogenic is conflicting, the class assigned by default is *“uncertain significance”*. As recently shown (Tavtigian et al., 2020), the ACMG/AMP guidelines are compatible with a quantitative Bayesian formulation, whose scaling as odd of pathogenicity allows an empirical calibration of the strength of the reported evidence.

A reference database, collecting annotated genetic variants by adopting the ACMG/AMP guidelines, is ClinVar (Landrum et al., 2020) which represents one of the main sources of information for gene classification in the ClinGen database (Section 1.1). ClinVar is a freely accessible, public archive, collecting reports of variants found in patient samples, assertions about their clinical significance, and other data, including the availability of supporting evidence. ClinVar thus allows users to infer relationships between human variations and the health status of the patients. Each variation has its own accession number, and, if multiple submitted records about the same variation/condition pair are present, they are aggregated under a single accession number. The adoption of a single variant identifier allows users to review all data submitted for a single variant, regardless of the condition for which it was interpreted. In fact, ClinVar neither curates content nor modifies interpretations associated with a single record. The alleles are reported according to the HGVS standards. Focusing on protein variants, the UniProt consortium is releasing a curated file reporting a list of protein variants, grouped in 3 classes: “*Disease”*, *“Polymorphism”* and *“Unclassified”* (humsavar UniProt, 2023). In the *humsavar* file, an OMIM identifier is associated with each pathogenic variant. Alternatively, the Human Gene Mutation Database (HGMD) is a proprietary database of mutations in human genes, associated with inherited diseases, which contains both inherited and somatic mutations (Stenson et al., 2020). The GWAS Catalog, supported by a collaborative initiative between the National Human Genome Research Institute and the EMBL-EBI, is another popular freely available database of SNP-trait associations, which can be easily integrated with other resources (Sollis et al., 2023).

The collection and curation of several variant databases is supported by ELIXIR, an intergovernmental organization that brings together bioinformatics resources for life sciences from across Europe (https://elixir-europe.org/). For example, the European Variation Archive (EVA) (Cezard et al., 2022) is an open-access database of all types of genetic variations (SNP, large structural variants, observed in germline or somatic sources) from all species. Submitted variants (in Variant Call Format, VCF) are merged, normalized and annotated for functional consequences and to determine allele frequency values in a study-specific manner. Human variants are also exchanged with dbSNP and other NCBI resources. DisGeNet (Piñero et al., 2020) is another database that integrates information on human gene-disease associations and variant-disease associations from different repositories. The data are annotated with controlled vocabularies and community-driven ontologies, and several original metrics are provided to assist the prioritization of genotype–phenotype relationships. Another ELIXIR core resource collecting information about variation is Ensembl Variation (Hunt et al., 2018), a resource linked to the Ensembl Genome Browser. It stores variants found in many species (including human) and, where available, associated diseases and phenotype information. Variant data are imported from a variety of sources (e.g., dbSNP) and subjected to a quality control process. They are then classified and their consequences predicted. Moreover, variant sets are created to help people retrieve a specific group of variants from a particular dataset. For human data, the linkage disequilibrium is also calculated for each variant, by population. A list of resources and databases for RD cited in this paragraph is reported in Supplementary Table S2.

## 3 Tools for rare disease genome interpretation

### 3.1 Automatic variant calling pipelines

The analysis of next-generation sequencing (NGS) experiments requires substantial bioinformatics resources. During the last years, a variety of analytical tools have been developed for the detection of genetic variants. Such tools assist all steps of the variant calling process, including quality control and trimming, alignment to the reference genome, identification, and annotation of SNVs and short indels. Although the Genome Analysis ToolKit (GATK) Best Practices guidelines define standard procedures for setting up a variant analysis pipeline, selecting the best approach among the variety of tools for NGS data processing can still be challenging. To overcome the issue and simplify the variant calling process, several “ready-to-use” bioinformatics pipelines to process ES and GS data have been made available. Some of them include: fastq2vcf (Gao et al., 2015), SeqMule (Guo et al., 2015), ExScalibur (Bao et al., 2015), Appreci8 (Sandmann et al., 2018), JWES (Ahmed et al., 2021), OVarFlow (Bathke and Lühken, 2021) and the recent DeepVariant (Poplin et al., 2018) that integrates a deep-learning-based variant caller. Most of those pipelines integrate many variant calling tools to increase sensitivity, but they are command-line applications to be installed on local servers. Alternatively, web-based options are available, e.g., Maser (Kinjo et al., 2018), CSI NGS Portal (An et al., 2020), and the most popular Galaxy (Afgan et al., 2018). Recently, *seqr*, a web tool for the analysis of rare disease genomes, has been made available by the Broad Institute (Pais et al., 2022). They are open-source platforms that provide a user-friendly graphical interface, improving the accessibility to computation analyses of genomic data. In particular, Galaxy users can freely create custom workflows or find already existing workflows, available on Galaxy Toolshed (Blankenberg et al., 2014), which can be run on public Galaxy servers. The disadvantages of using the web-based options are the limited amount of data that can be uploaded, the CPUs time, and the limitations on some tools on the public Galaxy platforms. However, Galaxy pipelines can also be run on a local Galaxy installation, or on a paid cloud infrastructure, e.g., Amazon cloud (AWS), using CloudMan (Afgan et al., 2010). Terra is another example of a web- and cloud-based platform, providing a compute environment to run optimized pipelines on Google Cloud. Galaxy, Terra, and other analysis components are integrated in a unified environment for data analysis and management, AnVIL (Schatz et al., 2022), designed to manage and store genomics data, enable population-scale analysis, and facilitate collaborative large-scale research projects. Nevertheless, “best practices” for variant calling in clinical settings, should be considered before choosing the most appropriate sequencing strategy, and the most reliable combination of tools for read alignment/preprocessing, variant calling and filtering (Koboldt, 2020).

Furthermore, to ensure the reproducibility of complex bioinformatics analysis, different workflow languages have been used to develop specific data analysis pipelines. The NextFlow core community (Ewels et al., 2020) collected a curated set of optimized procedures for the analysis of genomic data specific for rare disease. Similar projects include Dockstore (O’Connor et al., 2017), which provides containerized tools and workflows, currently supporting 4 different languages: the Workflow Description Language (WDL), Common Workflow Language (CWL), Nextflow, and Galaxy Workflows (GWs). Moreover, several workflows accessible on Dockstore can be easily launched in web-based platforms, such as Terra. These workflow languages are designed to handle some aspects of computational workflows, such as resources, software, and execution of analysis steps. Among those, Snakemake (Köster and Rahmann, 2012) and Nextflow (Di Tommaso et al., 2017) are commonly used for developing new research pipelines, while WDL and CWL workflows are preferred for large-scale projects (Reiter et al., 2021). Recently, a specific pipeline for the analysis of rare disease genome has been made available in NextFlow (Ewels et al., 2020).

Most of the above semi-automatic pipelines help streamline the generation of variant lists (in vcf format), but lack the downstream annotation and filtering steps that are necessary to identify disease-causing variants. To this end, different data-warehousing solutions to store genomic variants, along with the relevant genomic annotations, were deployed to allow a flexible and efficient data exploration. An example is GEMINI (Paila et al., 2013) and OpenCGA that supply the platform and the analysis framework to build customized genomic databases, to efficiently store data to be queried and visualized. A list of tools for variant calling and annotation is reported in Supplementary Table S3.

### 3.2 Gene prioritization tools

The objective of gene prioritization is to rank a large list of potential candidate genes based on their relevance for a disease. The prioritization algorithms identify the most promising genes, as to their association to the molecular basis of a given disease and/or a specific phenotype, for defining a therapeutic and/or diagnostic procedure. From the experimental point of view, the high-throughput techniques reduced the costs for generating a high amount of information about gene mutations. On the other hand, the identification of real links between genes and diseases is still a time- and money-consuming task. Therefore, the help of computational tools to reduce the number of genes to be investigated is strongly needed. Beyond the assumption that one gene codes for one function, the possibility that defects of one gene may be related to multiple diseases is now taken into account. At the same time, more genes can be involved in a given disease. In fact, a given metabolic pathway is composed of several protein functions, a defect in any of which may result in the pathway failure. Computational tools for gene prioritization use different sources of information to rank the candidate genes. Possible features are direct experimental data on gene sequences, mutations, expression (co-expression), gene-gene and protein-protein interactions, as well as more indirect evidence as ontologies, literature, information derived by model organisms. Different types of tools may differ by the focusing level (e.g., disease-specific or not), by the applied methodology (e.g., text-mining, similarity profiling, network analysis), by the approach to select the best candidate genes (e.g., ranking or filtering into smaller subsets), by the assumptions (i.e., genes may be directly or indirectly associated with a disease), or simply by the type of experimental evidence used for the analysis. Several works list the available tools on the basis of the state-of-the-art and classification applied (Moreau and Tranchevent, 2012; Piro and Di Cunto, 2012; Gill et al., 2014; Zolotareva and Kleine, 2019; Cabrera-Andrade et al., 2020; Jacobsen et al., 2022; Yuan et al., 2022). For instance, Jacobsen et al. applied phenotype-driven methods to improve diagnostic yields for RD, and listed 16 freely available tools (Jacobsen et al., 2022). Zolotareva and Kleine listed 14 tools, classifying them according to strategies, approach types, interfaces, input, and the types of evidence sources (Zolotareva and Kleine, 2019). Smedley and Robinson compared 7 tools and summarized their features in terms of exome input, types of variants analyzed, and approach (Smedley and Robinson, 2015). Problems related to long-term maintenance of academic software are very common (Jacobsen et al., 2022) and solutions have been proposed (Rother et al., 2012). A list of tools from the cited literature is reported in Supplementary Table S4. Among all gene prioritization methods, for instance, VarElect and ToppGene are part of standard diagnostic pipelines in the clinical settings. In particular, VarElect (Stelzer et al., 2016a) is a comprehensive, phenotype-dependent, variant/gene prioritization tool, based on the GeneCards suite (Stelzer et al., 2016b). The input of VarElect is a gene list together with a free-text phenotype description, such as disease and symptom terms, which represents a useful interface for non-skilled users. The tool prioritizes the genes on the basis of scores for the associated terms, computed on the appearance frequency in the entire GeneCards knowledgebase. The latter includes also the human disease database MalaCards (Rappaport et al., 2017), the human biological pathways of Pathcards (Belinky et al., 2015), and the gene expression information in cells and tissues of LifeMap Discovery (Edgar et al., 2013), for a total of 120 sources. The results of VarElect are displayed as a table of genes with decreasing phenotype relation scores. Alternatively, the gene prioritization task can be performed by ToppGene (Chen et al., 2007; Chen et al., 2009a; Chen et al., 2009b), a suite including tools for gene list functional enrichment, candidate gene prioritization, and identification and prioritization of novel disease candidate genes in the interactome. ToppGene selects genes in the training set on the basis of their association with disease, pathway, GO term, phenotype. The test set can be given by candidate genes from linkage analysis studies, differential expression in a particular disease or phenotype, interactome knowledge. The enrichment step is based on a variety of data sources that cover 14 types of annotation. For each type of annotation of each test gene, a similarity score is generated, by comparison to the enriched terms in the training set. The prioritized gene list is ranked on the aggregated values of the 14 similarity scores.

Finally, specific algorithms for the prioritization of RD-associated genes were recently developed (Zhu et al., 2012; Liu et al., 2017; Buphamalai et al., 2021; de la Fuente et al., 2023). Among them, for instance, an algorithm was developed, based on the calculation of a vertex-similarity score between each pair of genes, that was tested on a set of ∼1,600 known orphan disease-causing genes associated with 172 RDs (Zhu et al., 2012). Another method, which computes the topological similarity between genes connected in a PPI network, ranks the candidate genes combining two scores reflecting the local and global connectivity of the network (Liu et al., 2017). The success rate of this method can reach 50%–75% on a set of ∼1,200 genes collected from the Orphanet database. A more comprehensive approach evaluates the impact of rare gene defects, building a multiplex network with more than 20 million gene relationships organized into 46 network layers (Buphamalai et al., 2021). The analysis of 3,771 RDs reveals distinct phenotypic modules that can be used to accurately predict RD gene candidates. A recent tool (GLOWgenes), based on 33 functional networks classified in 13 knowledge categories, was able to recover genes associated with 91 genetic diseases classified into 20 families (de la Fuente et al., 2023). When applied to 15 unsolved cases, GLOWgenes was able to identify three new genes potentially associated with syndromic inherited retinal dystrophies.

### 3.3 Variant interpretation methods

Variant interpretation tools are *in silico* predictive programs that can help researchers in establishing the pathogenicity of the variations identified in the gene(s) of interest. Many approaches have been developed to perform these predictions, and their number has grown very rapidly in the last years. They mainly focus on predicting the impact of a missense variation on the structure and function of the associated protein, or on predicting effects on RNA splicing.

More recently, programs addressing more general noncoding variants have also been developed (Özkan et al., 2021). Researchers and clinicians tend to use variant interpretation tools in combination, as also suggested by the ACMG/AMP guidelines (Section 1.3). Nevertheless, their concordance in asserting the variant effects (especially of the predicted benign ones) has been rather low until present. More recently, however, newly developed algorithms have shown good performance in many types of genes and mutation mechanisms. Furthermore, by using gene-specific algorithms, and by calibrating them with well-characterized sets of benign and pathogenic variants, better results may be reached, than with general use algorithms (Ghosh et al., 2017).

In the last two decades, an impressive number of methods and algorithms for single amino acid substitution (SAS) have been devised to predict the variant effect on protein structure, function and interactions, to eventually identify those involved in molecular pathogenicity. As a matter of fact, SASs represent more than 40% of the unique variants found in the Exome Aggregation Consortium (Lek et al., 2016). Those methods are obviously not specific to RDs and have a broad range of applications (Capriotti et al., 2019; Katsonis et al., 2022; Pancotti et al., 2022). A selection of the most recent methods and resources is reported in Supplementary Table S5. Many of the early methods were based on the prediction of the effect of a single mutation on the protein thermodynamic stability, as destabilization is one of the key factors in pathogenesis (Capriotti et al., 2008; Dehouck et al., 2011; Worth et al., 2011; Fariselli et al., 2015; Laimer et al., 2015; Quan et al., 2016; Savojardo et al., 2016; Yang et al., 2018; Marabotti et al., 2020; Pires et al., 2020; Montanucci et al., 2022). Subsequent efforts and developments in the field produced last-generation methods, using one of three general strategies: i) prediction of the likelihood of a missense mutation for causing pathogenic changes in a protein (Sim et al., 2012; Adzhubei et al., 2013; Carter et al., 2013; Katsonis et al., 2014; Niroula et al., 2015; Capriotti et al., 2017; Raimondi et al., 2017; Rentzsch et al., 2019; Pejaver et al., 2020; Manfredi et al., 2022; Quinodoz et al., 2022); ii) evolutionary conservation analysis of the mutated sites; iii) methods combining different strategies (Stein et al., 2019; Petrosino et al., 2021). More recently, several methods have been developed to also predict the impact of variants in noncoding regions (Rojano et al., 2019; Katsonis et al., 2022; Tabarini et al., 2022). These methods include generic tools, which predict single-nucleotide pathogenic variants across the entire genome (Quang et al., 2015; Shihab et al., 2015; Zhou and Troyanskaya, 2015; Capriotti and Fariselli, 2017; Rentzsch et al., 2019) and more specific algorithms, which predict the impact of splicing variants (Desmet et al., 2009; Cheng et al., 2019; Jaganathan et al., 2019; Rentzsch et al., 2021). In particular, splicing-affecting variants are established contributors to RD, of which they may modulate the phenotypic outcome (Li et al., 2016; Scotti and Swanson, 2016).

**FIGURE 2:**
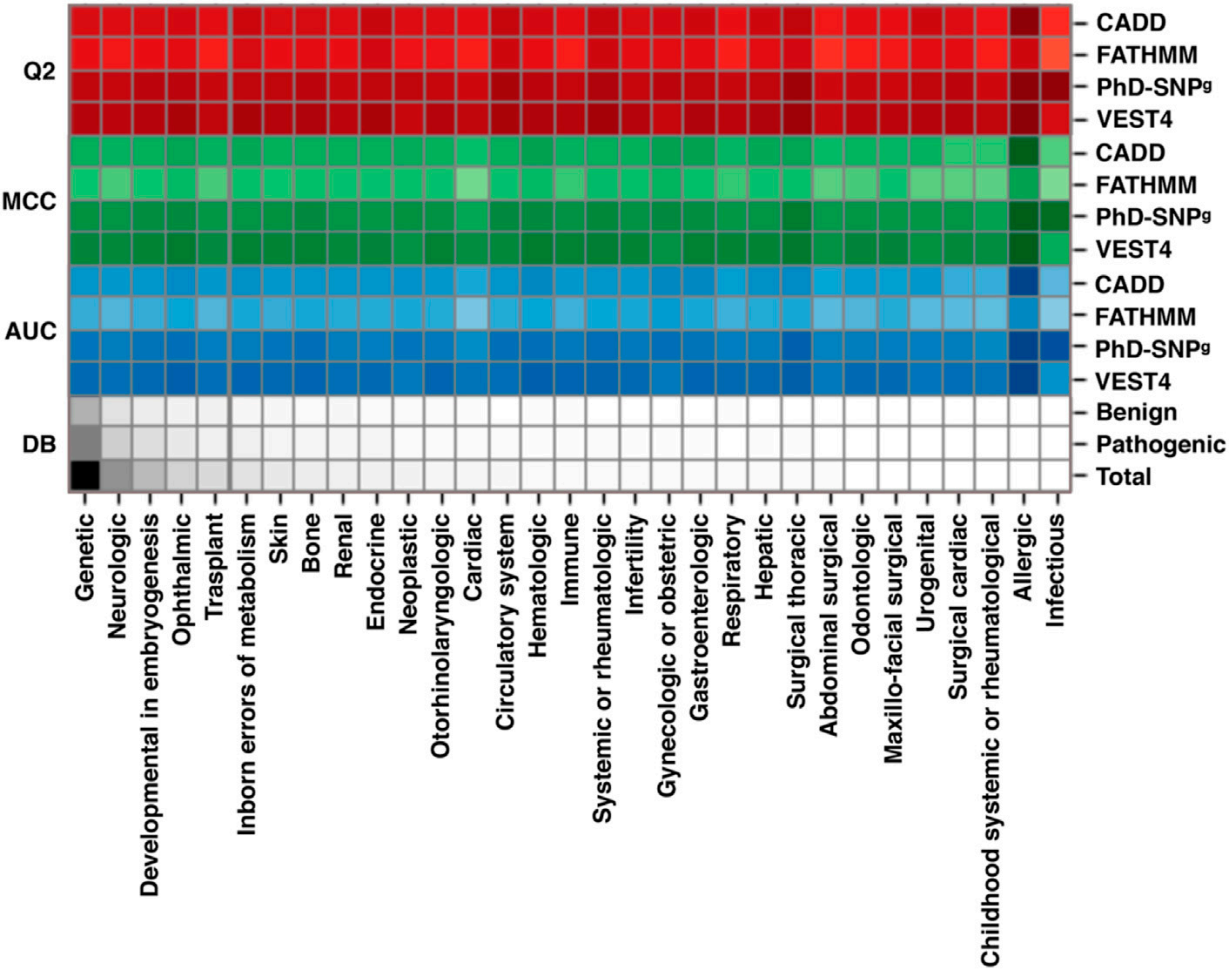
Performance of four state-of-the-art methods (CADD, FATHMM, PhD-SNP^g^ and VEST4) on a dataset of RD-associated variants from ClinVar database, featuring at least one annotation as Pathogenic or Benign. The scores are calculated for the different classes of RDs. All 27,648 variants (16,012 Pathogenic and 11,633 Benign) in our dataset are in the Genetic class. According to the Clinvar annotation, each variant can be classified in multiple RD groups. Performance parameters shown are: Q2, Overall Accuracy; MCC, Matthews Correlation Coefficient; AUC, Area Under the receiver operator characteristic Curve. DB indicates the fraction of each RD group in the dataset. The performance of CADD was calculated considering a Phred-like score threshold of 20. The color darkness in the drawing is proportional to the numerical values, which are reported in Supplementary Table S6. The predictions of the four methods are reported in Supplementary Materials.

To assess the performance of the available variant interpretation algorithms on the variants specifically associated with RDs, we collected a dataset of SAS from ClinVar (March 2022). Such a dataset (sas-rd-202203 in Supplementary Materials) is composed of ∼27,600 SAS in genes associated with rare genetic disorders from different RD classes. From RD-associated ClinVar genes, we selected 16,012 variants classified as *Pathogenic* and 11,633 *Benign*. The results of our analysis, scoring the performance of 4 state-of-the-art variant interpretation tools (CADD, FATHMM, PhD-SNP^g^ and VEST4), show that the selected methods reach on average 83% overall accuracy (Q2), 0.65 Matthews correlation coefficient (MCC), and >0.90 area under the ROC curve (AUC) (Supplementary Table S6). These results are in the same range of those reported in previous works, not limited to RDs (Capriotti and Fariselli, 2017; Benevenuta et al., 2021). A chromatic representation of the performance of the methods (Figure 2) reveals that VEST4 reaches the highest AUC (0.96) while FATHMM the lowest (0.83). Taking into account some possible data overfitting, we expect that the resulting measures of performance might be overestimated by no more than 2%–5% (Capriotti and Fariselli, 2017). The results of the four tools in predicting the effect of different RD classes exhibit some variation. Specifically, for the *Ophthalmic* RD class, with 7,889 variants (28.5%), the performance of the methods is slightly higher than average, reaching 85% overall accuracy, 0.69 Matthews correlation coefficient and 0.92 AUC. Conversely, the lowest performance was observed in predicting the impact of 2,152 variants associated with the *Cardiac* RD class (∼7.8%), with an overall accuracy below 80%, a Matthews correlation coefficient of 0.58, and an AUC of 0.87. Although our analysis shows that state-of-the-art methods for the prioritization of causative variants in RD-associated genes result in a high-performance level, further work is needed for improving the tools’ reliability, in view of the residual ∼10% of misclassified variants. In this regard, it appears that an important aspect to be considered for improving the predictions reliability is the conservation level at the variation site (Capriotti and Fariselli, 2022).

### 3.4 Genotype/phenotype association methods

Despite the progress in our capacity to prioritize disease-causing genotypes in clinical exomes and genomes, the large number of variants that remain to be evaluated for the diagnosis-making process is still a challenge. Computational analysis of phenotypes, in addition to genotypes, has proven powerful to improve the standardization and automation of NGS diagnostic pipelines from raw sequences to prioritized variants. The general principle, followed by such analyses, is to compute measures of similarity between the clinical manifestation in a patient and the description of disease(s) linked to a gene. Gene and/or variant prioritization tools measure ontological similarity between a set of query terms, representing the compendium of the patient’s clinical phenotypes, and the set of terms that are associated with any disease-gene (Smedley and Robinson, 2015). Algorithms underlying such tools have been developed, exploiting standardized collections of clinical terminologies, the most widely adopted of which is the Human Phenotype Ontology (HPO). The latter is used to assist clinical scientists and researchers in clustering and comparing phenotypes of patients with shared molecular background, with the aim to improve genetic diagnosis and genotype-to-phenotype correlations. Many tools that exploit the knowledge of known phenotypes of disease genes in humans and animal models have been developed. Such tools can be broadly categorized into two groups: those that take both phenotype and genotype data (VCF + HPO) as input, and those that only accept phenotype data (HPO only). These tools have been thoroughly reviewed and evaluated in a recent publication (Yuan et al., 2022). As an example, one of the earliest and most used tools is Exomiser (Robinson et al., 2014). Exomiser combines the most popular strategies for variant filtration with HPO to prioritize data in a VCF file. Despite its name, the Exomiser analysis framework is not limited to the exome but incorporates the Regulatory Mendelian Mutation (ReMM) score for relevance prediction of non-coding variations (SNVs and small InDels) (Smedley et al., 2016). In their review, Yuan et al. (2022) identified the two best performers in HPO-based gene prioritization to be LIRICAL (Robinson et al., 2020) and AMELIE (Birgmeier et al., 2020). Both of those recently published tools propose innovative and interesting analysis approaches. LIRICAL aims to overcome simple gene or variant ranking based on semantic similarity as a prioritization scheme, by introducing a likelihood-ratio test to provide an estimate of the post-test probability of candidate diagnoses. AMELIE, conversely, consists in an end-to-end machine learning approach with web interface, that finds relevant literature supporting the disease causality of genetic variants and their association with different clinical presentations. In the benchmark from Yuan et al. (2022), the two methods often resulted in quite different predictions of highly ranked causal genes, and such a complementarity suggests a possible integrative approach to further enhance the diagnostic efficiency. In a recent work, genotype/phenotype association methods were tested on a set of 4,877 molecularly diagnosed cases, affected by RDs, from the 100,000 Genome Project (Jacobsen et al., 2022). On this set, Exomiser was able to recall 82% and 92% of the diagnosed cases as the top hit, and within the top 5 scores, respectively. These positive results are going to render phenotype-genotype association tools essential for RD diagnosis in the clinical routine.

## 4 Use cases on rare disease genome interpretation

**FIGURE 3:**
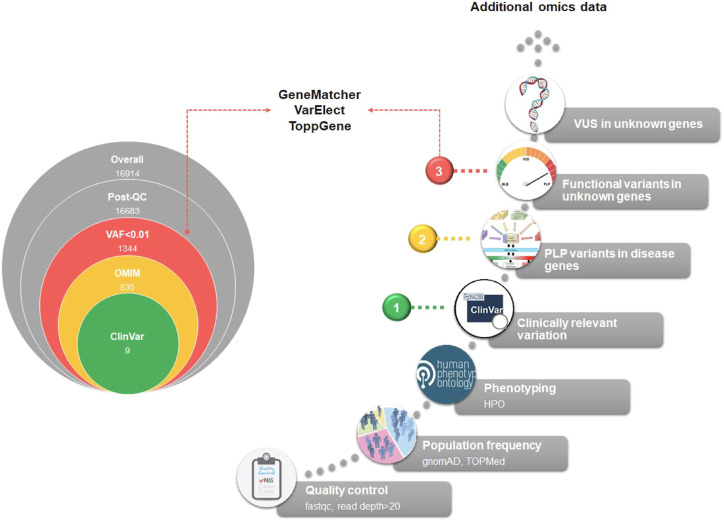
Exome analysis flowchart. A diagram of the main steps of NGS data analysis is shown. On the left, the progressive reduction by filtering in the number of likely disease-causing variants is shown, for a general patient case. The reported numbers are from a typical single patient case. On the right, the filtering process is detailed. Based on the identified variants, we can recognize three different diagnostic situations: (1, green dot) identification of P/LP variants with well-established association to RD phenotype; (2, yellow dot) identification of new P/LP variants in genes with known association to the phenotype; (3, red dot) identification of functional variants in genes with unknown association with the phenotype. A fourth case should be considered, i.e., the identification of VUS variants in genes with unknown association with the phenotype. In this case, complementing different approaches, such as short-read genome sequencing with RNA sequencing, and methyl profiling, should be considered to elucidate the molecular mechanism of the disease and improve the diagnostic yield.

**FIGURE 4:**
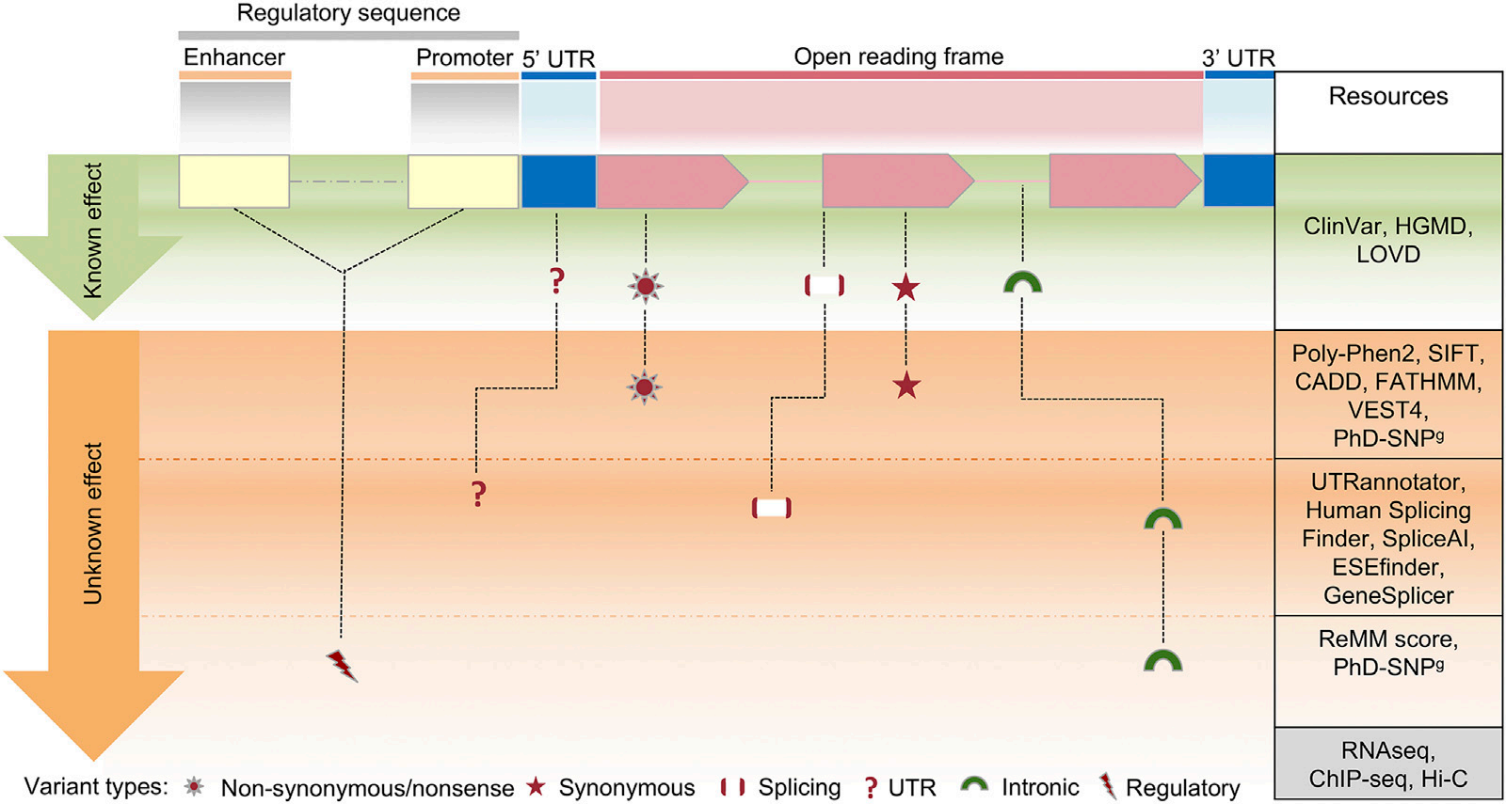
Schematic view of the clinical variant interpretation process. In a human protein-coding gene, a variant in the exons of an open reading frame can result in synonymous or nonsynonymous changes, while a variant in other areas (splice or intronic regions) can impact on splicing regulation. Changes within regulatory sequences (yellow and blue) can affect transcription and translation regulation of gene expression. On the right column, a selection of the most commonly used resources for variant interpretation is reported, distinguished by their gene location. Several methods are currently available to predict the effect of coding variants, however the interpretation of variants in deep-intronic regions or in regulatory elements is still challenging, due to the limited number of *in silico* prediction approaches. Such shortcomings can be overcome by parallel sequence analysis of the whole exome/genome together with multi-omics technologies, including RNA sequencing (transcriptome analysis), ChIP-seq (chromatin immunoprecipitation assay) and HiC (high-throughput chromosome conformation capture).

The diagnostic workflow of NGS genetic testing is composed of three levels of data analysis: i) quality control of raw data, ii) variant annotation, and iii) variant filtering. On the basis of the annotation level of the variants detected after the variant calling procedure, we can identify three main steps of analysis. In Figure 3 we summarized the main filtering steps, including the approximate number of unique variants that can be identified in a single subject after a clinical exome. In order to efficiently prioritize clinically relevant variations among all types of captured variants described in Figure 4, we need to adopt different analytical strategies. Several resources, including databases of genomic variation and phenotypes, population frequency data and *in silico* prediction approaches, can be used for the interpretation of each type of variant (Supplementary Table S5).

According to the variant types, distinguished by their gene location and the currently available resources for variant interpretation, we can define the three following possible cases.

### 4.1 Identification of pathogenic variants associated with RD phenotypes

In this case, the analysis workflow is well-defined and relatively easy. In the hypothetical case shown in Figure 4, after the application of the filters of variant allele frequency (VAF) and phenotyping (see below), known clinically relevant variants in disease genes that fit with the phenotype are selected to be reported.

The VAF is retrieved from population databases (Section 1.3), using resources that aggregate exome and genome sequencing data from a variety of large-scale sequencing projects, and make summary data available for the wider scientific community. They include sequencing data of both affected and unaffected subjects or different populations (Gudmundsson et al., 2022). In current diagnostic settings, ultra-rare and rare variants (VAF < 0.001 and VAF < 0.01, respectively), as well as private variants (not annotated in population databases) are selected. Of course, this primary filter can be modified according to the prevalence in the population of a specific disorder. Thus, in some diagnostic settings, also low-frequency variants (VAF < 0.05) can be selected (Andolfo et al., 2021).

The exact characterization of the phenotype (*“phenotyping”*) is one of the most relevant aspects of NGS genetic testing, and it is often considered a major challenge for the NGS-based genetic diagnosis. Generally, *phenotyping* is obtained using a standardized vocabulary of phenotypic abnormalities encountered in human disease, such as that provided by HPO database (Section 2.4). Clinically relevant variants can be prioritized using public repositories reporting correlation between genetic variants and phenotypes, such as ClinVar and HGMD (Section 1.3; Supplementary Table S2).

### 4.2 Identification of pathogenic variants in RD associated genes

Herein, after the application of the aforementioned filters of variant frequency, phenotyping, and clinically relevant variants in disease genes, no variants are prioritized. In this case, to prioritize pathogenic/likely pathogenic (PLP) variants associated with the phenotype, ACMG/AMP guidelines for variant interpretation (Section 1.3) are used.

According to those guidelines, the pathogenicity of each variant is evaluated by gathering evidence from various sources: population data, computational and predictive data, functional data, and segregation data. Computational and predictive data are obtained by using several *in silico* prediction programs described in Section 2.3. Those tools are mainly devoted to the evaluation of the missense variants, which constitute a major set of VUS (Variant of Unknown Significance). The ACMG/AMP guidelines recommend complete concordance of predictions among all *in silico* algorithms used, without specifying the number or types of algorithms. However, many studies have provided rules for the classification of non-synonymous variants based on the integration of different prediction tools (Ghosh et al., 2017; Li et al., 2019; Nicora et al., 2022).

For phenotype characterization, the analysis of splicing variants is also relevant. The prioritization of splice site variants can be performed by web server tools, such as Human Splicing Finder (Desmet et al., 2009), MMSplice (Cheng et al., 2019), SpliceAI (Jaganathan et al., 2019) and CADD-splice (Rentzsch et al., 2021), that can highlight potential splicing-affecting variants outside the canonical splicing sites. ACMG/AMP variant classification can be achieved in such cases by using InterVar or wInterVar (Li and Wang, 2017), a web server that enables user-friendly variant interpretation with both an automated interpretation step and a manual adjustment step. Functional data that supports the pathogenic effect of newly discovered variants is not typically included in the standard diagnostic process of NGS genetic testing. Nevertheless, laboratories with an extensive experience in a specific disease area, can provide additional functional evaluation for new variants as part of their diagnostic protocols (Thouvenot et al., 2016; Ellingford et al., 2022).

Finally, segregation and allele data are fundamental to correctly assess the pathogenicity of variants. For this reason, in diagnostic settings the trio analysis, i.e., the combined genomic analysis of patient and parents, is strongly recommended (Alfares et al., 2020; French et al., 2022; Gabriel et al., 2022).

### 4.3 Identification of functional variants in genes with unknown RD association

Currently, the diagnostic process reaches a definitive diagnosis only in about 50% of the cases, leaving many patients with strongly-suspected genetic diseases without molecular explanations. In such cases, all variants with potential functional effects on any gene must be considered, under the hypothesis that the pathogenic role of the causative gene is still unknown. The initial filtering steps, similar to the previous scenarios, consist of removing all variants unlikely to be implicated in the disease, either because of low quality in exome or high frequency in population. Very stringent frequency thresholds are used, since it is likely that the considered disease is extremely rare. Then, the pedigree is analyzed to maintain only the variants that co-segregate with the phenotype according to any Mendelian transmission model. The variant-affected genes are prioritized to remove those that show a high grade of variability in the general population and to highlight those with a plausible biological role in the disease phenotype. The resulting set of genes with functional variants, poor population variability and biological compatibility is released in gene matching tools to search for other patients who are affected by alterations in the same genes (Section 2.2). Once a ‘match’ occurs, the researchers are connected through the system and can share molecular and clinical details of the patients, potentially concluding that they are both affected by the same disease, caused by the matched genes.

An example of successful gene-matching regards a 19-years-old girl seen at Federico II University Hospital, Naples, Italy. The girl was affected by a severe clinical picture, composed of complex brain malformations, extraocular muscle anomalies, severe intellectual disability, and drug-refractory epilepsy. Despite the presentation strongly suggesting an underlying genetic cause, thorough molecular and metabolic investigation failed to yield any plausible explanation. The patient was, then, enrolled in the Telethon Undiagnosed Diseases Program (TUDP) and underwent patient-parent trio ES. Variant filtering and manual revision did not find causative variants but highlighted those in four non-disease genes (PLEKHN1, NR5A2, TMEM89, DHX37). The patient’s clinical and molecular descriptions were released in PhenomeCentral for gene-matching (Buske et al., 2015; Sobreira et al., 2017; Osmond et al., 2022), where only for DHX37 a consistent match with other patients with syndromic intellectual disability was found (Paine et al., 2019).

However, depending on disease type and patient selection, exome sequencing has been estimated to lead to a diagnosis in 30%–50% of rare Mendelian diseases (Frésard and Montgomery, 2018). A recent analysis shows that 14% of the recent diagnoses could be successfully performed by the combination of automatic and research approaches, looking for variants occurring in genomic regions poorly covered by exome sequencing (100,000 Genomes Project Pilot Investigators et al., 2021). Thus, the whole genome sequencing approach is becoming more relevant for the diagnosis of rare disorders (Turro et al., 2020). Accordingly, a large variety of computational approaches have been recently developed to score the impact of variants in noncoding regions (Shihab et al., 2015; Zhou and Troyanskaya, 2015; Ioannidis et al., 2016; Ionita-Laza et al., 2016; Capriotti and Fariselli, 2017; Rentzsch et al., 2019; Wells et al., 2019). In addition, for the interpretation of these potentially regulatory variants, the simultaneous and integrated use of multiple layers of omics technologies, such as whole-genome and transcriptome sequencing, is also increasingly being considered (Hasin et al., 2017; Kerr et al., 2020).

We expect that such methods will soon become the reference diagnostic tools in clinical settings. In this direction, a recent work describes approaches and discusses strategies for the diagnosis of rare and undiagnosed diseases, based on the analysis of the whole genome (Marwaha et al., 2022).

## 5 Data sharing and FAIRification

In the context of RDs, data sharing between institutions and across countries is crucial for maximising the potential of the generated genomic data (Saunders et al., 2019). It allows for the recruitment of larger cohorts of patients, thereby increasing statistical, and diagnostic, power. Sensitive RD patient data are collected by multiple institutions, whose registries are always difficult to aggregate. Sharing such data is essential for the development and maintenance of large databases, which are essential for federated analysis and discovery. In this context, the guiding principles of Findable, Accessible, Interoperable and Reusable (FAIR) data for humans and computers (Wilkinson et al., 2016) were developed, to ensure responsible sharing of health data and safeguarding of subjects. Since 2014, when “FAIR” acronym was first coined, and, because of their potential, FAIR principles have been widely endorsed by the RD community, the International Rare Diseases Research Consortium (IRDiRC) and the ELIXIR research infrastructure. In fact, adopting FAIR principles allows researchers and clinicians to integrate data from different resources in compliance with the restrictions of data accessibility, and thus answer questions involving multiple resources. For example, many types of genomic data, including features linked to the genomic coordinates of a reference genome, are always difficult to locate and access. A recent application of the FAIR principles to genomic data allowed the development of a track search service, which integrates metadata from various hubs, by adopting a set of recommendations for genomic data sharing (Gundersen et al., 2021). In addition, tools and pipelines developed for the analysis of genomic data, such as those described in this review, undoubtedly fall in the category of “research software”, which is now considered part of FAIR by the European Commission. Indeed, FAIR principles can be applied not only to data, but to research software as well (Jiménez et al., 2017; Lamprecht et al., 2020).

A recent initiative, aiming at making FAIR (‘FAIRification’) 24 ERN (European Reference Networks) registries of RD patients, allowed collecting ninety-eight critical FAIRification challenges and proposing solutions to address them (Dos Santos Vieira et al., 2022). Awareness of the FAIRification challenges learned from initiatives like this one, which are strongly supported by the ELIXIR community, plays an important role in identifying solutions aimed at harmonizing RD data. Nevertheless, most resources collect unique data and there are wide differences in content, format, and language across them. This heterogeneity makes it virtually impossible to harmonize data from different resources, wasting the time and effort of data analysts and compromising any large-scale project aimed at improving RD research and supporting RD patients. It is therefore critical to put effort in the FAIRification process, both for humans and machines, so that data (including registries) can be queried in an unambiguous, global and federated way.

Inline with this need, the ELIXIR bio. tools portal (Ison et al., 2019) provides a comprehensive registry of software and databases that facilitates the search, understanding, use, and recognition of biomedical scientific resources. Among the 27,471 tools available on the portal, we identified 303 tools that are part of the *“Rare Diseases”* collection, domain, or topic and refer to a total of 165 functions described with the semantic terms of the EDAM ontology (Ison et al., 2013). After reviewing a list of 303 RD tools, we integrated them with other bio. tools methods, to develop a curated set of core resources for analyzing rare disease genomes.

The resources and tools collected in Supplementary Tables S2–S5 have been evaluated according to five criteria, related to their accessibility, update status, number of citations, and development stage (reported with “mature” tag in bio. tools). This type of evaluation, which assigns a score ranging from 1 to 5, represents a step toward the establishment of a standardized protocol for their clinical application.

## 6 Conclusions and future directions

Quick and accurate diagnosis are key issues for public health in general and for RDs in particular. The diagnostic delay for many RDs may at present reach up to decades (Molster et al., 2016; Heuyer et al., 2017), with an average time of about 4–5 years (Yan et al., 2020). In the journey towards diagnosis (also named the “*diagnostic odyssey*”) patients may receive misdiagnosis and consequent inappropriate treatments and care. Diagnostic delay and misdiagnosis are due to many factors: RDs are infrequent, thus it is difficult to achieve a critical mass of data; data are sensitive, heterogeneous (clinical data, patient registries, variants, etc.) and usually fragmented (different communities and efforts collect data on specific RDs of interest using different formats, schemas, etc.) with poor interoperability, and a single, comprehensive repository for RDs does not exist.

In recent years, the development of new tools and resources, and the advances in data sharing practices and integrated analyses have allowed to reach an appropriate diagnosis for a sizable proportion of patients (Marwaha et al., 2022). Indeed, combining data from different sources, and using computational tools to analyze them in an integrated manner, is crucial to validate candidate variants, identify disease causative genes, perform genotype-phenotype associations, and elucidate the underlying molecular mechanism of a disease.

However, RD patients and expertise are still very scattered from each other, and knowledge and data sparsity, fragmentation, heterogeneity and poor interoperability often make integration and sharing of information extremely difficult if not impossible. Moreover, RD data are sensitive and recent technologies and practices gave rise to the further challenge of reconciling the benefits of data sharing and integration with privacy protection and ethical issues. Indeed, one of the major challenges nowadays consist in the implementation of reliable procedures for improving data sharing and the development of standardized tools and pipelines to enable reproducible research, while at the same time guaranteeing privacy rights.

To address these challenges, many international consortia have been established to create and integrate global infrastructure for RD research. At the European level, Solve-RD (solving the unsolved RDs, (Zurek et al., 2021), and RD-Connect (Lochmüller et al., 2018) enabled the creation of interdisciplinary teams to actively share and jointly analyze existing patient’s data. These initiatives leverage existing computational infrastructures to share registries and standardize data among clinicians and scientists. In particular, the RD-Connect consortium promoted the development of the Genome-Phenome Analysis Platform (GPAP) (Laurie et al., 2022), and its integration with the PhenomeCentral (Osmond et al., 2022) and DECIPHER (Foreman et al., 2022). The GPAP platform facilitates the collation, discovery, sharing, and analysis of standardized genome-phenome data within a collaborative environment.

In this context, the implementation of a FAIR ecosystem of federated resources is essential for boosting research and diagnosis by decreasing RD data fragmentation and increasing data quality, with great advantages also in terms of time saving and sustainability. Although the developers and maintainers of the major RD resources and tools are already moving in the direction of FAIR data and software sharing, much still remains to be done to achieve the systematic application of FAIR principles by all players of the ecosystem, including data providers, data stewards and managers, software developers, researchers and clinicians, patients associations, research institutions, hospitals, and infrastructures. The transparent access to data and tools by the scientific community is recognized nowadays as one of the major challenges for improving RD diagnosis.

## Supplementary material

SUPPLEMENTARY TABLE S1 Analysis of the disease types in the Orphanet database.

SUPPLEMENTARY TABLE S2 Databases of ontologies, gene/protein networks and variants.

SUPPLEMENTARY TABLE S3 Tools for variant calling and annotation.

SUPPLEMENTARY TABLE S4 Resources for gene prioritization.

SUPPLEMENTARY TABLE S5 Resources and tools for variant interpretation.

SUPPLEMENTARY TABLE S6 Performance of 4 methods in the prediction of rare disease associated variants.

Supplementary Data Predictions of CADD, FATHMM, PhD-SNP^g^ and VEST4 on the dataset of 27,468 rare disease associated variants (sas-rd-202203) from 2,697 genes.
